# Strengthening care for emergencies: what is the level of awareness and utilization of Emergency Medical Services (EMS) in FCT, Nigeria?

**DOI:** 10.1186/s12873-024-00991-2

**Published:** 2024-04-29

**Authors:** Sunday Eke Nto, Toluwani Oluwatola, Olugbemisola Samuel, Hilary Okagbue, Sunday Atobatele, Andikan Ibanga, Abdullahi Adegoke, Doubra Emuren, Saidu Ahmed Dumbulwa, Sidney Sampson, Saheed Dipo Isiaka, Salamatu Sadiq

**Affiliations:** 1 Sydani Institute for Research and Innovation, Abuja, Nigeria; 2 Sydani Group, Abuja, Nigeria; 3 National Emergency Medical Service and Ambulance System, Abuja, Nigeria

**Keywords:** Emergency Medical Services, Utilization, Awareness, Emergency Care, Public perception

## Abstract

**Background:**

As part of the Federal Government of Nigeria’s desire to increase medical coverage among the citizenry, the National Emergency Medical Service and Ambulance Scheme (NEMSAS) was set up and piloted in the FCT in 2022. To gauge the progress so far, this study sought to assess the level and determinants of public awareness and utilization of Emergency Medical Services (EMS) among residents of the Federal Capital Territory, Abuja.

**Methods:**

A cross-sectional study was conducted in June 2023 among 1177 respondents residing in FCT Abuja at the time of the survey. Data was collected by trained research assistants using an interviewer-administered questionnaire and purposive sampling was adopted. The level of awareness and the socio-demographic determinants of the level of awareness in the FCT were assessed. Logistic regression was used to find predictors of EMS awareness and utilization.

**Results:**

57.8% of respondents are aware of EMS, while 42.2% are not. 62.7% are uncertain about the source of information for EMS with only a minority relying on word of mouth (17.7%), traditional media (11.1%), or social media (8.5%). 91.4% have not accessed or utilized EMS via the toll-free emergency line, while only 8.6% reported doing so. There are median EMS awareness and utilization differences across gender, age, location, and employment status of the respondents. Additionally, the multivariate logistic regression showed that age, location, and employment status are significant predictors of EMS awareness and utilization. Males have lower odds of awareness and utilization of EMS compared to females. Furthermore, there was a significant relationship between EMS access and utilization (Chi-square = 80.748, *p* < 0.001). However, awareness did not necessarily translate to utilization.

**Conclusion:**

The relationship between EMS awareness and utilization and the unmasked predictors in this paper should be factored into the design of interventions to increase access and utilization of EMS in Nigeria.

**Supplementary Information:**

The online version contains supplementary material available at 10.1186/s12873-024-00991-2.

## Background

A medical emergency is an acute injury or illness that poses an immediate risk to an individual’s life or long-term health [1]. They range from self-resolving allergic reactions to cardiovascular emergencies and life-threatening injuries [2]. Emergencies contribute significantly to the burden of diseases globally, contributing to 41.7% of morbidities and 50.7% of all mortalities [1]. In Nigeria, medical emergencies range from acute severe malaria to neurological injuries following trauma and contribute to 345 DALYs per 1000 to the burden of diseases in the country [3, 4].

Most emergencies require assistance from a medical service provider, such as emergency medical services [5]. Emergency Medical Service (EMS) is a “*comprehensive system that provides the arrangements of personnel, facilities, and equipment for the effective, coordinated, and timely delivery of health and safety services to victims of sudden illness or injury*” [6]. EMS aims to provide the first point of contact of urgent medical care to satisfactorily treat the presenting conditions, from the scene of the incidents to the point of definitive care to victims of sudden or life-threatening emergencies [7, 8]. This prevents unwarranted mortality or other preventable bodily infractions [9, 10]. There is a wide spectrum of emergency care services - pre-hospital, hospital, and rehabilitative care and the linkages between the components, including but not limited to emergency personnel, an emergency communication system, emergency infrastructure, an integrated emergency ambulance service system, emergency equipment, and a functional trauma system in the receiving facility [11].

The emergency care system indirectly impacts mortality and morbidity at all levels of society [12, 13]. Hence, emergency care is essential to the health system as it is the first point of contact for many injured, including those in road crashes worldwide [7, 14]. However, timely emergency care is not readily available or prohibitively expensive in some regions of the world [7]. It has been estimated that more than 50% of deaths and more than 30% of disabilities in low- and middle-income countries could be addressed by effective emergency care [15].

The provision of EMS in Nigeria is still nascent after different failed attempts by national and state governments at establishing a comprehensive emergency system in the country. This has resulted in inadequate and often delayed medical care for individuals with medical emergencies [16–18]. Challenges of EMS include a lack of infrastructure, limited resources, and inadequate training [16]. Although improvements in emergency care in Nigeria will involve strengthening human resources, physical resources, and organization and planning for prehospital care and care in hospitals (which are all supply-driven), there is an even greater need to ensure that the citizens are aware of and utilize these supplies when they are available, especially as communication has been noted as a major driver of an effective EMS system [19]. Given this, it is important to understand the level of awareness of the availability of emergency medical services in FCT.

The National Health Act of 2014 made financial provisions to fund EMS in Nigeria to address these challenges and improve the provision of EMS in Nigeria. This led to the creation of the National Emergency Medical Service and Ambulance Scheme (NEMSAS), which is tasked with the responsibility of organizing Emergency Medical Services and Pre-hospital and Emergency care services and ensuring that these services have a sustainable and high-impact service element in Nigeria’s health system architecture. NEMSAS piloted the provision of its services in Nigeria’s Federal Capital Territory (FCT) by leveraging the national emergency call toll line (112) for the provision of EMS to the residents.

We surveyed the residents of Nigeria’s FCT to understand their level of awareness of the availability of EMS. The specific objectives were to:

i. Assess the level of public awareness and utilization of EMS in FCT.
ii. Assess the relationship between awareness and utilization of EMS in FCT.
iii. Predict key determinants of awareness and utilization of EMS using demographic variables.

## Methods

### Study population and design

This was a cross-sectional study conducted in June 2023 among 1177 residents of FCT, Abuja. The Federal Capital Territory (FCT) is a federal jurisdiction situated in the heart of Nigeria. It is home to Abuja, the nation’s capital city and the seat of government with an estimated population of 3,067,500 persons [20]. The Federal Capital Territory comprises six Area councils: Abaji, Abuja Municipal Area Council (AMAC), Bwari, Gwagwalada, Kuje, and Kwali, collectively covering an area of approximately 7,290 square kilometres [21].

Purposive sampling was used to assess residents’ level of awareness of the availability of EMS using an interviewer-administered questionnaire (included as **supplementary material**). There were two main reasons for choosing purposive sampling.

- **Absence of Census Data**: One significant reason for adopting purposive sampling was the absence of reliable census data. The last census in Nigeria was conducted in 2006, making it outdated.
- **Specific Criteria**: Secondly, purposive sampling allowed researchers to select respondents based on specific criteria relevant to the study objectives. In this case, the criterion was that residents must have been living in the FCT since 2021. This targeted residents who would have had the opportunity to be aware of NEMSAS launched in 2022.

The original sample size of 1300 was obtained and 123 representing 9.5% were excluded because of nonresponse and missing data yielding the final sample size of 1177 that was analyzed. The details of the sample size determination is presented in Table 1 and the population data was obtained from [20].

**Table 1:** Sample Size Determination

Location	Population	Initial Sample Size	Final Sample Size
Abaji	127,900	54	47
AMAC	1,693,400	717	632
Bwari	500,100	212	181
Gwagwalada	346,000	147	134
Kuje	212,100	90	103
Kwali	188,000	80	80
**Total**	**3,067,500**	**1300**	**1177**

AMAC: Abuja Municipal Council

This research used purposive sampling and assigned samples based on the proportion of the projected population of the FCT. This is quite different from quota sampling because the criteria were based on residency in Abuja since 2021. Unlike quota sampling, we are not interested in a sample reflecting the characteristics of the population in terms of certain attributes, such as age, gender, or socioeconomic status.

Research assistants were recruited to survey under the supervision of experts in survey methodology. Kobo Collect was the data management tool used in data collection.

### Reliability test and piloting

The contents of the questionnaire was reviewed by two of the co-authors who are experts in survey and monitoring and evaluation. Afterwards, a pilot study was carried out in New Karu Town in Karu local government area of Nasarawa State which shares boundaries with FCT. The area was chosen because majority of the residents worked in the FCT. The limitation of the pilot study was most of the respondents work in AMAC and their views are very similar.

The feedback from the pilot study were reviewed by experts and incorporated into the questionnaire and to ensure that the items adequately cover the construct they intend to measure and are relevant to the research objectives.

Cronbach’s Alpha, which is a measure of reliability, could not be performed because there are very few items, and most are not Likert-scaled.

### Variables

The questionnaire was divided into two sections. Section A contains the four demographic variables namely: gender, age, location (the six area councils), and employment status. Section B contains variables that assess the degree of knowledge of EMS. The first variable is to assess if the respondents have been in an emergency and the second one is to gauge their respective responses during emergencies. The third variable is to determine if the respondents are aware of the toll-free emergency line that can be used to utilize the EMS offered. The fourth variable is to determine the source of information about EMS and the fifth variable is to ascertain if the respondents know of anyone that has accessed EMS. The sixth variable contained the type of emergency reported, and the final variable was to determine if the respondents benefited from first aid or basic life-supportive training in the past.

### Data analysis

The data was extracted from Kobo Collect and transferred to Microsoft Excel for extensive data cleaning. SPSS (Statistical Package for the Social Sciences) version 27 was used for descriptive statistics, crosstabulation, Chi-square test, Mann-Whitney test, and Logistic regression. R software version 4.3.2 was used for Kruskal-Wallis. A *p*-value less than 0.05 is considered significant except otherwise stated.

### Ethical considerations

Ethical approval was obtained from the National Health Research Ethics Committee (NHREC) with ref number: NREC/01/01/2007-16/05/2023. Informed consent was also obtained from all participants in the study and were duly informed of their inalienable rights to withdraw participation from the study at any point in the study. Additionally, assent was sought and received from participants aged less than 18 years old, while consent was received from their parents and legal guardians.

## Results

### Demographic analysis

The summary of the demographic characteristics of the respondents is presented in Table 2. 43.1 per cent were female, 35.8% were between 31 and 45 years and 53.7% were interviewed in AMAC and 59.7% were employed.

**Table 2:** Summary of the demographic variables

Variable	Frequency	Percentage
**Gender**		
Female	507	43.1
Male	670	56.9
**Age**		
11–15	131	11.1
16–30	391	33.2
31–45	421	35.8
46–60	198	16.8
Above 60	36	3.1
**Location**		
Abaji	47	4.0
AMAC	632	53.7
Bwari	181	15.4
Gwagwalada	134	11.4
Kuje	103	8.8
Kwali	80	6.8
**Employment**		
Student	248	21.1
Unemployed	226	19.2
Employed	703	59.7

### Measures of awareness and other attributes

The survey showed that 80.8% have witnessed or been in emergencies. The data reveals, as shown in Table 3, diverse responses to emergencies, with 33.6% of individuals opting to call for help, 17.2% providing first aid, and 35.3% did nothing in response to the emergency. Some chose to contact family members (8.5%), while a smaller percentage directly called an emergency helpline (4.0%). Additionally, 1.4% of individuals used their smartphones to document or record the situation. 57.8% of respondents in the dataset are aware of EMS, while 42.2% are not. Most respondents (62.7%) are uncertain about the source of information for EMS with only a minority relying on word of mouth (17.7%), traditional media (11.1%), or social media (8.5%). The majority (91.4%) have not accessed or utilized EMS via the toll-free emergency line, while only 8.6% reported doing so. 79.4% have not reported any emergency, 8.8% have reported fire incidents and 7.4% and 4.5% have reported road accidents and medical emergencies respectively.

**Table 3:** Summary of measures of awareness and other attributes

Variable	Frequency	Percentage
**Ever witnessed or been in an emergency**		
No	226	19.2
Yes	951	80.8
**Response to emergency**		
Call for help	395	33.6
Provide first aid	202	17.2
Did nothing	416	35.3
Phone family member	100	8.5
Call the emergency helpline	47	4.0
Document or record using a smartphone	17	1.4
**Awareness of EMS**		
No	497	42.2
Yes	680	57.8
**Source of Information for EMS**		
Word of mouth	208	17.7
Traditional media	131	11.1
Social media	100	8.5
Don’t know	738	62.7
**Accessed EMS via toll-free emergency line**		
No	1076	91.4
Yes	101	8.6
**Type of emergency reported**		per cent
Fire	103	8.8
Road accident	87	7.4
Medical emergency	53	4.5
None	934	79.4
**Ever had any form of first aid or basic life-supportive training**		
No	815	69.2
Yes	362	30.8

### Differences in the awareness

Mann-Whitney test (MWT) showed there is a significant median EMS awareness between males and females. The other three analyses as presented in Table 4 were done using the Kruskal-Wallis test (KWT) which showed significant median EMS awareness across age, location, and employment status of the respondents.

**Table 4:** Demographic Differences in the Awareness Level

Variable	No	Yes	MWT /KWT
**Gender**			
Female	231	276	159,891*
Male	266	404	
**Age**			
11–15	71	60	24.48**
16–30	155	236	
31–45	157	264	
46–60	89	109	
Above 60	25	11	
**Location**			
Abaji	24	23	59.63**
AMAC	306	326	
Bwari	58	123	
Gwagwalada	70	64	
Kuje	17	86	
Kwali	22	58	
**Employment**			
Student	101	147	24.3**
Unemployed	128	98	
Employed	268	435	

*P < 0.05: **p < 0.01; MWT = Mann-Whitney Test; KWT = Kruskal-Wallis Test

### Predictors of EMS awareness

Binary logistic regression was used to predict EMS awareness using the four demographic variables. The univariate models showed that all four demographic variables are significant predictors of EMS awareness while all, but gender were significant predictors in the multivariate model presented in Table 5.

**Table 5:** Predictors of EMS Awareness

Variable^1^		Univariate models		Multivariable model^2^	
		Crude OR (95% CI)	*p*-value	Crude OR (95% CI)	*p*-value
Gender	Female	1	0.044	1	0.125
	Male	0.79 (0.62, 0.99)		0.82 (0.64, 1.06)	
Age	11–15	1	< 0.001	1	**0.001**
	16–30	1.92 (0.87, 4.22)		0.97 (0.40, 2.38)	
	31–45	3.46 (1.66, 7.24)		2.07 (0.94, 4.59)	
	46–60	3.82 (1.83, 7.98)		**2.53 (1.15, 5.57)**	
	Above 60	2.78 (1.30, 5.97)		1.88 (0.83, 4.25)	
Location	Abaji	1	< 0.001	1	**< 0.001**
	AMAC	0.36 (0.17, 0.77)		**0.36 (0.17, 0.79)**	
	Bwari	0.40 (0.24, 0.68)		**0.38 (0.22, 0.65)**	
	Gwagwalada	0.80 (0.45, 1.44)		0.79 (0.43, 1.44)	
	Kuje	0.35 (0.19, 0.63)		**0.33 (0.18, 0.62)**	
	Kwali	1.92 (0.94, 3.92)		2.01 (0.97, 4.19)	
Employment	Student		< 0.001		**< 0.001**
	Unemployed	0.90 (0.67, 1.20)		1.23 (0.82, 1.84)	
	Employed	0.47 (0.35, 0.64)		**0.49 (0.35, 0.68)**	

^1^The first listed category of each variable was taken as reference value ^2^Following multiple logistic regression, variables significantly associated with EMS awareness are presented in bold

In both models, males have lower odds of being aware of EMS compared to females. Additionally, respondents aged 31 and above have higher odds of awareness compared to those aged between 11 and 15. Those who are employed have lower odds of being aware of EMS compared to students.

### Differences in utilizing EMS

Mann-Whitney test (MWT) showed there is a significant median EMS utilization between males and females. The other three analyses as presented in Table 6 were done using the Kruskal-Wallis test (KWT) which showed significant median EMS utilization across age, location, and employment status of the respondents.

**Table 6:** Demographic Differences in UtilizingEMS

Variable	No	Yes	MWT /KWT
**Gender**			
Female	471	36	165427.5*
Male	605	65	
**Age**			
11–15	130	1	21.537**
16–30	367	24	
31–45	375	46	
46–60	173	25	
Above 60	31	5	
**Location**			45.83**
Abaji	41	6	
AMAC	587	45	
Bwari	173	8	
Gwagwalada	127	7	
Kuje	77	26	
Kwali	71	9	
**Employment**			17.84**
Student	239	9	
Unemployed	214	12	
Employed	623	80	

### Predictors of EMS utilization

Binary logistic regression was used to predict EMS utilization using the four demographic variables. The univariate and multivariate models revealed that all but gender of the demographic variables are significant predictors of EMS awareness as presented in Table 7.

**Table 7:** Predictors of EMS Utilization

Variable^1^		Univariate models		Multivariable model^2^	
		Crude OR (95% CI)	*p*-value	Crude OR (95% CI)	*p*-value
Gender	Female	1	0.116	1	0.570
	Male	0.71 (0.47, 1.09)		0.88 (0.56, 1.37)	
Age	11–15	1	0.003	1	**0.046**
	16–30	**0.05 (0.01, 0.42)**		**0.04 (0.00, 0.42)**	
	31–45	0.41 (0.15, 1.14)		**0.26 (0.08, 0.83)**	
	46–60	0.76 (0.28, 2.05)		0.40 (0.13, 1.22)	
	Above 60	0.90 (0.32, 2.52)		0.44 (0.14, 1.38)	
Location	Abaji	1	< 0.001	1	**< 0.001**
	AMAC	1.15 (0.38, 3.48)		0.83 (0.27, 2.59)	
	Bwari	0.61 (0.28, 1.29)		**0.44 (0.20, 0.96)**	
	Gwagwalada	0.37 (0.14, 0.98)		**0.28 (0.10, 0.78)**	
	Kuje	0.44 (0.16, 1.22)		**0.32 (0.11, 0.91)**	
	Kwali	2.66 (1.17, 6.07)		2.12 (0.90, 5.01)	
Employment	Student		< 0.001		**0.005**
	Unemployed	0.29 (0.15, 0.59)		0.52 (0.22, 1.20)	
	Employed	0.44 (0.23, 0.82)		**0.33 (0.16, 0.66)**	

^1^The first listed category of each variable was taken as reference value ^2^Following multiple logistic regression, variables significantly associated with EMS Utilization are presented in bold

### Relationship between EMS awareness and utilization

The Chi-square test showed a significant relationship between EMS awareness and utilization (Chi-square = 80.748, *p* < 0.001). The Chi-square was computed from the contingency table and the Fisher test was not carried out since all the expected frequencies are greater than 5, despite reporting a zero in one of the cells of the observed frequency **(**Table 8**)**.

**Table 8:** Contingency Table of the relationship between EMS awareness and utilization

		EMS utilization		Total
		No	Yes	
**EMS Awareness**	No	497	0	497
	Yes	579	101	680
Total		1076	101	1177

## Discussion

Findings from the study revealed that the awareness of EMS in the FCT is slightly above average. This corroborates with the findings from another study of awareness of EMS in Nigeria which found that less than half of the respondents were aware of any form of emergency medical response in Nigeria [22]. However, this contrasts with the findings of a study that assessed the awareness of emergency response services among users of EMS in FCT and Keffi [21], which showed more than 90% of users of EMS in Abuja were aware of emergency medical response service and emergency call numbers. The differences in the level of awareness and the one previously conducted in the FCT may be due to the difference in our study population composition. This paper surveyed residents as opposed to beneficiaries of EMS.

Within the FCT, there is also significant variation in the level of awareness among the area councils in the FCT. AMAC seems to be doing better in terms of the level of awareness when compared with other area councils. The probable reasons for these variations could be differences in urbanization, population density, level of communication, outreaches done in the area council, and historical emergency incidents in the area council. These factors have been noted as major drivers of awareness of emergency medical services in the literature [14, 22]. The study also found a statistically significant difference in the level of awareness by gender. The finding is in alignment with other studies conducted in Nigeria, which have reported that women are less aware of EMS than men [23]. This finding is unsurprising given the gender disparities in the access to healthcare services in Nigeria [24]. However, our logistic regression predicts that males are less likely to be aware of EMS. Among the different age groups, significant statistical differences exist. More precisely, older age groups are more likely to be aware of EMS compared to the youngest group (11–15). This finding is consistent with the findings of [25] that health literacy is associated with senior citizens as they may be dealing with multiple health challenges that require constant visits to the hospital.

Again, students are more likely to be aware of EMS compared to employed and unemployed individuals since students had a higher odds ratio than both the employed and unemployed participants. This finding is intuitively appealing as students are expected to be more aware because they are often in educational environments where they may come across information about emergency services as part of their curriculum or through educational programs. Again, students may be more connected to various sources of information, including educational materials, news, and online resources, which could increase their awareness of EMS. Furthermore, students might be more active on social media and have a lifestyle that exposes them to a variety of information, including public safety and emergency services, community activities, volunteer work, internships, etc.

From Table 8, among individuals aware of EMS, 14.8% utilized the service, indicating a positive association. Notably, among those without EMS awareness, no instances of utilization were observed. This absence hints at a potential link between awareness and utilization, emphasizing the impact of awareness campaigns on encouraging EMS use. The probable reason for low utilization can be low confidence in the EMS as the low utilization of the primary health care services has been attributed to low confidence [26].

Additionally, the Mann-Whitney test indicates a substantial gender difference, with more males (65) utilizing EMS compared to females (36). This point to a potential gender-related disparity in emergency healthcare-seeking behaviour. This finding deviates from the findings of other studies that have noted the prevalence of healthcare utilization among male users [27, 28]. Hence, our logistic regression predicts that males have lesser odds of utilizing the EMS than their female counterparts.

The Kruskal-Wallis test exposes significant age-related variations, with higher EMS utilization in the age groups 31–45 and 46–60, suggesting that middle-aged individuals are more likely to seek emergency medical assistance. This finding is partially in alignment with other studies that found a positive relationship between healthcare utilization and age [29].

Location-based differences are evident, as the Kruskal-Wallis test highlights varying EMS utilization across different areas. Kuje exhibits the highest utilization, while Abaji and Gwagwalada show comparatively lower usage, indicating potential discrepancies in access or awareness. Different areas had varying odds of EMS utilization, indicating a geographical influence on the likelihood of utilizing EMS services. The probable reason for this can be the nearness of health facilities as proximity has been highlighted as a key determinant for the utilization of healthcare services [30]. The nature of the composition of the population could also be responsible.

Moreover, employment status significantly influences EMS utilization, with employed individuals (80) demonstrating the highest usage, followed by unemployed (12) and students (9). This emphasizes the impact of occupational factors on emergency healthcare-seeking behaviour. Those that are employed are likely to have health insurance or have finances to address any health emergencies. Also the distance travelled for work and prolonged absence from residence as a result of work increases their odds of soliciting for EMS. These findings are in tune with [31] who noted that occupational differences or characteristics of individuals have a significant influence on healthcare service utilization. However, this contrasts with the findings of [32] who reported that the unemployed had higher odds of using formal healthcare facilities than those currently employed. The difference in geographical scope of these studies, urban, compared to the slum can be the probable reason for the variance.

### Limitation of study

Our findings may have been affected by social desirability bias as many of our respondents might have answered the questions in a way that they believed was socially acceptable or desirable, rather than reporting their true behaviours. This can happen because people want to avoid appearing uninformed, irresponsible, or in a negative light. Consequently, this can lead to overestimation of the level of awareness in the study. People might also overestimate their knowledge of EMS, especially if they feel they should know more about them. Additionally, it may lead to underreporting of utilization as some people may be reluctant to admit usage of EMS. To mitigate the effect of the limitation, the following strategies were deployed. First, the respondents were given assurances that their responses would remain anonymous and confidential. This helped reduce the fear of judgment, encouraging more honest answers. Second, the research assistants were trained to be neutral and non-judgmental in their interactions with respondents. Lastly, some of the foreseeable issues were addressed after piloting.

## Conclusion

The study concluded that the level of awareness and utilization of EMS in FCT is slightly above average, and awareness does not necessarily lead to utilization. The findings from this study have important policy implications. Primarily, there is an evident need for expansive awareness campaigns to enlighten the public about the existence of EMS, with a specific emphasis on promoting the 112 emergency number. Equally, there is a need to drive gender equity in EMS awareness and utilization. Notably, the prominence of word of mouth as an information source emphasizes the importance of maintaining a high degree of responsiveness and quality of care within the EMS system, as positive experiences become a driving force for referrals. In crafting future campaign strategies, it is imperative to consider the diverse awareness landscapes across different area councils. Tailoring communication channels to the specific contexts of each location will be paramount for effective outreach.

### Electronic supplementary material

Below is the link to the electronic supplementary material.

Supplementary Material 1

## Data Availability

The data is not publicly available but can be provided upon reasonable request from the corresponding author.

## Abbreviations

FCT: Federal Capital Territory, Abuja
AMAC: Abuja Municipal Area Council
SPSS: Statistical Package for the Social Sciences
NHREC: National Health Research Ethics Committee
MWT: Mann-Whitney test
KWT: Kruskal-Wallis test

## References

[1] Ramanayake RPJC, Ranasingha S, Lakmini S (2014). Management of emergencies in general practice: role of general practitioners. J Family Med Prim care.

[2] Woyessa AH, Dibaba BY, Hirko GF, Palanichamy T. (2019). Spectrum, Pattern, and Clinical Outcomes of Adult Emergency Department Admissions in Selected Hospitals of Western Ethiopia: A Hospital-Based Prospective Study. *Emergency Medicine International*, *2019*, 8374017 10.1155/2019/8374017. 10.1155/2019/8374017 PMC6701330 31467720

[3] Okobia MN, Osime U (2003). The spectrum of emergencies in an accident center in Benin-city. Nigeria Sahel Med J.

[4] Oparah SK, Philip-Ephraim E, Williams U (2010). Spectrum of medical emergencies presenting at the University Teaching Hospital, Calabar. Mary Slessor J Med.

[5] Ramanayake RPJC, Ranasingha S, Lakmini S (2014). Management of emergencies in general practice: role of general practitioners. J Family Med Prim care.

[6] Garrone M (2011). Prehospital ultrasound as the evolution of the Franco-German model of prehospital EMS. Crit Ultrasound J.

[7] World Health Organization. Advocating for emergency care: a guide for nongovernmental organizations. World Health Organization; 2022.

[8] Federal Ministry of Health. (2016). Policy on Emergency Medical Services (EMS) in Nigeria. Retrieved from: https://nesgroup.org/download_policy_drafts/EMS%20Policy%202016_1661866073.pdf.

[9] World Health Organization. (2010); *Strengthening care for the injured: success stories and lessons learned from around the world*. World Health Organization. Accessed form: https://www.who.int/publications/i/item/strengthening-care-for-the-injured-success-stories-and-lessons-learned-from-around-the-world.

[10] Rathore N, Jain PK, Parida M. A sustainable model for emergency medical services in developing countries: a novel approach using partial outsourcing and machine learning. Risk Manage Healthc Policy. 2022;193–218. 10.2147/rmhp.s338186. 10.2147/RMHP.S338186 PMC8841749 35173497

[11] Saberian P, Shafiee A, Hasani-Sharamin P, Rafiemanesh H, Baratloo A (2023). The General Public Awareness of Emergency Conditions and the services provided by Emergency Medical Services. Bull Emerg Trauma.

[12] Burkle FM (2019). Challenges of global public health emergencies: development of a health-crisis management framework. Tohoku J Exp Med.

[13] Kelen, G. D., Wolfe, R., D’Onofrio, G., Mills, A. M., Diercks, D., Stern, S. A., …Sokolove, P. E. (2021). Emergency department crowding: the canary in the health care system. *NEJM Catalyst Innovations in Care Delivery*, *2*(5).

[14] Aljabri D, Albinali H (2022). Public awareness and use of 997 emergency medical service phone numbers during the COVID-19 pandemic. Front Public Health.

[15] Reynolds TA, Sawe H, Rubiano AM, Do Shin S, Wallis L, Mock CN. Strengthening health systems to provide emergency care. Disease Control Priorities: Improving Health Reducing Poverty. 2017;3rd edition. 10.1596/978-1-4648-0527-1_ch13. 30212151

[16] Oyedokun TO, Islam EM, Eke NO, Oladipo O, Akinola OO, Salami O (2023). Out of hospital emergency care in Nigeria: a narrative review. Afr J Emerg Medicine:Revue africaine de la Med D’urgence.

[17] Venkatraman C, Odusola AO, Malolan C, Kola-Korolo O, Olaomi O, Idris J, Nwariaku FE (2021). Lagos state ambulance service: a performance evaluation. Eur J Trauma Emerg Surg.

[18] Usoro A, Aiwonodagbon B, Strong J, Kivlehan S, Akodu BA, Olufadeji A (2021). Perspectives on the current state of Nigeria’s emergency care system among participants of an emergency medicine symposium: a qualitative appraisal. BMJ open.

[19] Kironji, A. G., Hodkinson, P., De Ramirez, S. S., Anest, T., Wallis, L., Razzak,J., … Hansoti, B. (2018). Identifying barriers for out-of-hospital emergency care in low and low-middle income countries: a systematic review. *BMC Health Services Research*, *18*(1), 1–20. DOI: https://doi.org/10.1186/s12913-018-3091-0. 10.1186/s12913-018-3091-0 PMC5907770 29673360

[20] City Population. (2022). Population of FCT Abuja. (Online) https://citypopulation.de/en/nigeria/admin/NGA015__federal_capital_territory/.

[21] Mac PA, Kroeger A, Airiohuodion PE (2019). Needs assessment of emergency medical and rescue services in Abuja/Nigeria and environs. BMC Emerg Med.

[22] Yonemoto N, Kada A, Yokoyama H, Nonogi H (2018). Public awareness of the need to call emergency medical services following the onset of acute myocardial infarction and associated factors in Japan. J Int Med Res.

[23] Akinreni T, Filani T, Asuni O, Owodunni F. Awareness and utilization of emergency response service to road traffic crashes in Nigeria: a cross-sectional study. J Public Health Emerg. 2023;7. 10.21037/jphe-23-31.

[24] Ulasi I (2008). Gender bias in access to healthcare in Nigeria: a study of end-stage renal disease. Trop Doct.

[25] Kuyinu YA, Femi-Adebayo TT, Adebayo BI, Abdurraheem-Salami I, Odusanya OO (2020). Health literacy: prevalence and determinants in Lagos State. Nigeria PloS One.

[26] Nwokoro UU, Ugwa OM, Ekenna AC, Obi IF, Onwuliri CD. Determinants of primary healthcare services utilization in an under-resourced rural community in Enugu State, Nigeria: a cross-sectional study. Pan Afr Med J. 2022;42(1). 10.11604/pamj.2022.42.209.33317. 10.11604/pamj.2022.42.209.33317 PMC9548321 36258898

[27] Michael, G. C., Aliyu, I., Andesati, A., Grema, B. A., Musa, A. M., Abu, R. B., …Edighotu, E. (2019). Utilization of accident and emergency department at a semi-urban Nigerian hospital: a preliminary prospective study. Journal of Acute Disease, 8(3),106–112. DOI: https://doi.org/10.4103/2221-6189.259109.

[28] Usar IJ, Mairiga BB (2020). Access and Utilization of Healthcare Services at the University of Jos Health Centre Jos, Plateau State, Nigeria. J Epidemiol Soc Nigeria.

[29] Mobosi IA, Okonta PO, Ameh CA (2022). Socio-economic determinants of demand for healthcare utilization in Nsukka Local Government Area of Enugu State Nigeria. Afr Social Sci Humanit J.

[30] Obiechina GO, Ekenedo GO. Factors affecting utilization of university health services in a tertiary institution in South–West Nigeria. Niger J Clin Pract. 2013;16(4). 10.4103/1119-3077.116888. 10.4103/1119-3077.116888 23974738

[31] Archibong EP, Bassey GE, Isokon BE, Eneji R. Income level and healthcare utilization in Calabar metropolis of Cross River state. Nigeria Heliyon. 2020;6(9). 10.1016/j.heliyon.2020.e04983. 10.1016/j.heliyon.2020.e04983 PMC7522490 33015387

[32] Fayehun, O., Ajisola, M., Uthman, O., Oyebode, O., Oladejo, A., Owoaje, E., … Improving Health in Slums Collaborative. (2022). A contextual exploration of healthcare service use in urban slums in Nigeria. Plos one, 17(2), e0264725.DOI: https://doi.org/10.1371/journal.pone.0264725. 10.1371/journal.pone.0264725 PMC8880927 35213671

