# Supplementary material for: Strengthening care for emergencies: what is the level of awareness and utilization of Emergency Medical Services (EMS) in FCT, Nigeria?
Source: BMC Emerg Med. 2024 Apr 29;24:73. doi: 10.1186/s12873-024-00991-2 (PMC11057177; doi:10.1186/s12873-024-00991-2)
Supplement: Supplementary file 1 — Supplementary Material 1 [file 12873_2024_991_MOESM1_ESM.docx]

**Supplementary**

We are carrying out this study to understand the level of awareness and utilization of Emergency Medical Services (EMS) in FCT, Nigeria. Your participation in this survey is invaluable, as it contributes to improving the effectiveness of Emergency Medical Services (EMS) and enhancing emergency care systems in FCT, Abuja, Nigeria. Thank you for taking the time to share your experiences and perspectives. Your contribution is greatly appreciated.

**SECTION A**

**Personal Data:**

1. **Gender:** Male ( ) Female ( )

2. **Age**: 11 – 15 ( ) 16 – 30 ( ) 31 – 45 ( ) 46 – 60 ( ) Above 60 ( )

3. **Location**: Abaji ( ) AMAC ( ) Bwari ( ) Gwagwalada ( ) Kuje ( ) Kwali ( )

4. **Employment**: Student ( ) Unemployed ( ) Employed ( )

**SECTION B**

1. **Have you ever witnessed or been in an emergency?** No ( ) Yes ( )
2. **What was your response to such an emergency?** Call for help ( ) Provide first aid ( ) Did nothing ( ) Phone family member ( ) Call the emergency helpline ( ) Document or record using a smartphone ()
3. **Are you aware of Emergency Medical Services (EMS)?** No ( ) Yes ( )
4. **How did you hear about Emergency Medical Services (EMS)?** Word of Mouth ( ) Traditional Media ( ) Social Media ( ) Don’t know ( )
5. **Have you ever accessed Emergency Medical Services (EMS) via a toll-free emergency line?** No ( ) Yes ( )
6. **What type of Emergency did you report?** Fire ( ) Road accident ( ) Medical Emergency ( ) None ( )
7. **Have you ever had any form of first aid or basic life-supporting training?** No ( ) Yes ( )
